# ASINA Project: Towards a Methodological Data-Driven Sustainable and Safe-by-Design Approach for the Development of Nanomaterials

**DOI:** 10.3389/fbioe.2021.805096

**Published:** 2022-01-28

**Authors:** Irini Furxhi, Massimo Perucca, Magda Blosi, Jesús Lopez de Ipiña, Juliana Oliveira, Finbarr Murphy, Anna Luisa Costa

**Affiliations:** ^1^ Transgero Limited, Limerick, Ireland; ^2^ Department of Accounting and Finance, Kemmy Business School, University of Limerick, Limerick, Ireland; ^3^ PROJECT-SAS, Faenza, Italy; ^4^ National Research Council, Institute of Science and Technology for Ceramics, Faenza, Italy; ^5^ TECNALIA Research and Innovation—Basque Research and Technology Alliance (BRTA), Parque Tecnológico de Alava, Miñano, Spain; ^6^ CeNTI—Centre of Nanotechnology and Smart Materials, Vila Nova de Famalicão, Portugal

**Keywords:** safe-by-design, sustainable-by-design, artificial intelligence, digital twins, nanotechnology

## Abstract

The novel chemical strategy for sustainability calls for a Sustainable and Safe-by-Design (SSbD) holistic approach to achieve protection of public health and the environment, industrial relevance, societal empowerment, and regulatory preparedness. Based on it, the ASINA project expands a data-driven Management Methodology (ASINA-SMM) capturing quality, safety, and sustainability criteria across the Nano-Enabled Products’ (NEPs) life cycle. We base the development of this methodology through value chains of highly representative classes of NEPs in the market, namely, (i) self-cleaning/air-purifying/antimicrobial coatings and (ii) nano-structured capsules delivering active phases in cosmetics. These NEPs improve environmental quality and human health/wellness and have innovative competence to industrial sectors such as healthcare, textiles, cosmetics, and medical devices. The purpose of this article is to visually exhibit and explain the ASINA approach, which allows identifying, combining, and addressing the following pillars: environmental impact, techno-economic performance, functionality, and human and environmental safety when developing novel NEPs, at an early stage. A metamodel supports the above by utilizing quality data collected throughout the NEPs’ life cycle, for maximization of functionality (to meet stakeholders needs) and nano-safety (regulatory obligations) and for the minimization of costs (to meet business requirements) and environmental impacts (to achieve sustainability). Furthermore, ASINA explores digitalization opportunities (digital twins) to speed the nano-industry translation into automatic progress towards economic, social, environmental, and governance sustainability.

## 1 Introduction

Due to the ongoing ambiguity around the potential adverse effects of nanomaterials (NMs) and their nanoforms on humans and the environment, the Safe-by-Design (SbD) concept was introduced in the emerging field of nanotechnology (Kraegeloh et al., 2018). Its implementation initiated with the European (EU) projects SanoWork NanoMICEX and Scaffold and was further developed by ProSafe and NanoReg2 (Soeteman-Hernandez et al., 2019). Since then, a number of ongoing EU projects are focused on the SbD notion, to mention a few, ASINA^1^ SAbyNA^1^, SABYDOMA^2^, SbD4Nano^3^, HARMLESS^4^, and SUNSHINE^5^. In addition, other activities have been created around the safety aspects of NMs such as the Nanomaterials Expert Group^6^, the Malta Initiative^7^, and the EU NanoSafetyCluster^8^.

Despite the fact that conceptions of SbD coexist, they all have the same purpose, to assess and increase safety in the development of NMs or Nano-Enabled Products (NEPs) as early as feasible into the production phase (Bottero et al., 2017; Schwarz-Plaschg et al., 2017). However, how to effectively manage safety issues at the product development stage and allow the selection of the best SbD solution is still being researched. Because of the uncertainty of NMs’ negative effects, the lack of defined guidelines, quality data (Schmutz et al., 2020), and a risk governmental framework (Trump et al., 2020), it has been difficult to bring them to market, and as a result, their potential advantages are underutilized. Another hurdle is the multidisciplinarity of the stakeholders involved in this challenge. Industry, regulators, and academia with different roles and needs are collided into the sphere of accelerating innovation within societal and ethical grounds. As there is no systematic Sustainable and SbD approach (SSbD) in place for NEPs’ life cycle (LC) and not all actors are experienced in considering safety, there is a need for a methodological approach enabling all actors to connect and consider all essential aspects of NEPs early during product development (Schmutz et al., 2020).

To fill this gap, within the ASINA project, we elaborate a data-driven SSbD Management Methodology (ASINA-SMM) by (1) taking up and extending the principles of the SbD approach developed for nanotechnologies, (2) involving industrial actors responsible for its uptake, (3) bringing digital technologies to allow digitization of nanomanufacturing, and (4) applying optimization and decision support algorithms in design and re-design stages for a continuous NEPs performance improvement through their LC. The approach is built, trained, and learned on two value chains of NEPs: self-cleaning/air-purifying/antimicrobial coatings and nano-structured capsules delivering active phases in cosmetics. The aim of this perspective paper is:

1) to present the extensions of the early phase of the NMs/NEPs design process, from the primary SbD concept^9^ to the SSbD one, and
2) to describe the ASINA-SMM metamodel, its approach, and implementation through the ASINA-Expert System (ASINA-ES) tool.

### 1.1 Extensions From the Primary SbD Approach

The primary SbD approach can be applied in different industries (e.g., paints and textiles) or used by regulators as a reference tool (Kraegeloh et al., 2018; Soeteman-Hernandez et al., 2019). The main elements of this approach are as follows: (1) it uses a stage-gate innovation approach; (2) it is based on three pillars: *Safe materials* and *products*, *Safe production,* and *Safe use and end-of-life*; (3) it includes actions for maximizing safety while maintaining functionality; and (4) it is integrated into a *Safe Innovation Approach* (see description in (Kraegeloh et al., 2018; Soeteman-Hernandez et al., 2019).

The stage-gate model (Cooper, 2008) describes the consecutive steps of information gathering and decision-making during the innovation process from idea to launch, and is applied to structure the SbD concept along the development of NM/NEP. ASINA-SMM moves towards a SSbD approach, by including the economic and environmental sustainability, in particular.

-ASINA-SMM is based on a multi-circular stepwise approach allowing continuous improvement of NEPs functional, environmental, economic, and safety performance. Indeed, ASINA-SMM is addressed to industrial applications, and it comprises an iterative six-sigma-inspired process based on DMADV main steps: Define, Measure, Analyze, Design, and Verify, in order to better represent the reality of production stages (Hjorth et al., 2017). The iterations are necessary to build up knowledge for the data-driven approach (Giubilato et al., 2020).

- ASINA-SMM aims at setting a replicable general model, which is implemented through the ASINA-ES, whose architecture allows performing the SSbD also for other NMs, NEPs, and applications with respect to the Value Chains considered within ASINA.

- ASINA-SMM can be integrated into business management models that promote continuous improvement—such as Six Sigma or ISO management systems—to deploy the SSbD concept within the general process of “Design and development of products and services” managed by these models.

-The ASINA-SMM is also based on the three pillars, extended to match the scope of the topic at hand. In the first pillar, ASINA adds lower cost and greener synthesis procedures by considering different materials and process design hypothesis (Ortelli et al., 2019). In the second, ASINA-SMM explores novel digital technologies and artificial intelligence for safe production while maintaining operational excellence (Ortelli et al., 2020; López De Ipiña et al., 2021). In the third, ASINA-SMM merges life cycle assessment (LCA) and life cycle costing (LCC) in order to ensure the safety, sustainability, and cost-effectiveness (Perucca and Benveniste, 2010; Oreto et al., 2021).

-The ASINA-SMM is embedded into and sets the frame for a roadmap, a simple guideline for improving NEPs in a data-driven way, providing a trusted environment, in combination with regulatory preparedness (Soeteman-Hernandez et al., 2019). The roadmap will provide the state-of-the-art scientific knowledge by artificial intelligence using FAIRified data (the process that assures Findability, Accessibility, Interoperability, and Reusability of the generated data) (Furxhi, 2022), specific methods for the production of NEPs, characterization, and testing with relevant regulatory endpoints (Botte et al., 2021). FAIR data are based on FAIR principles; these FAIR principles acquired popularity in the scientific community and are expected to grow into a cornerstone of research policy and requirements for research data management plan (Dunning et al., 2017). The principles emphasize a number of important preconditions for data sharing, asking researchers to consider the prospect of future data sharing and reuse from the start (Boeckhout et al., 2018). Their simplicity and flexibility are significant assets, allowing for the formation of agreed goals and courses of action in research data management. As a result, the FAIR principles give a vital stimulus to a data-driven research culture, allowing for transparent data reuse for accelerating nanotechnology research (Tropsha et al., 2017; Boeckhout et al., 2018). However, individual researchers and research organizations, as well as research communities, will have to rise to the occasion.

### 1.2 The Approach

#### 1.2.1 ASINA-SMM

**FIGURE 1:**
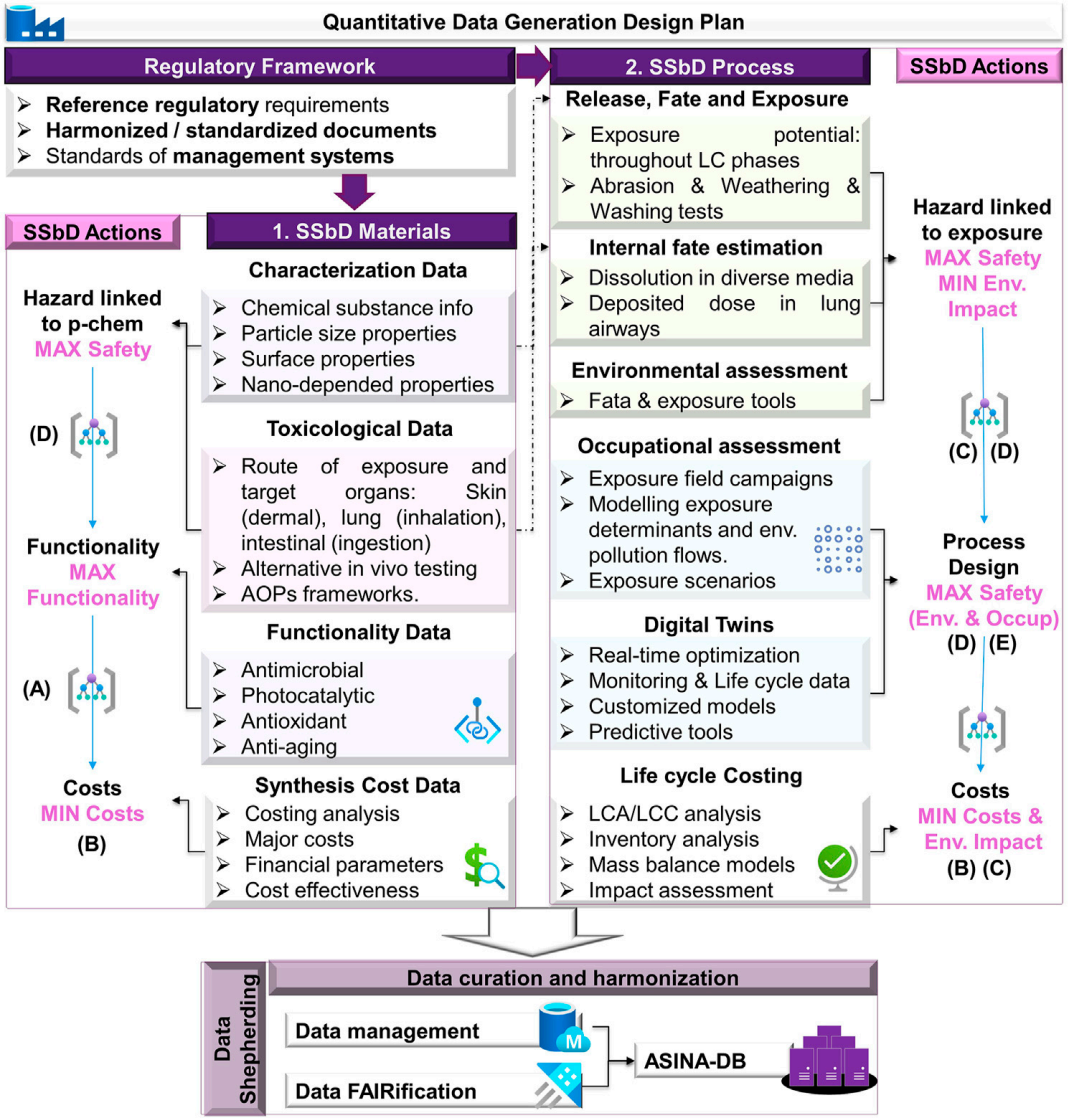
The ASINA-SMM data generation plan considering **(A)** functionality, **(B)** cost-effectiveness, **(C)** environmental sustainability, and **(D)** nano-safety while exploring digital technologies **(E)**.

The ASINA-SMM is a quantitative data-driven approach based on a robust data generation plan for the SSbD implementation to the production of NEPs, as presented in Figure 1. The holistic approach integrates safety, circularity, and functionality of materials, products, and processes throughout their LC while minimizing environmental footprint. It aims to facilitate the transition to a safe, carbon-neutral, and resource-efficient industrial ecosystem. For this purpose, four dimensions in the design of new NEPs are considered: (A) functionality, (B) cost-effectiveness, (C) environmental sustainability, and (D) nano-safety (Figure 1). Each LC phase is analyzed to define specific and independent design cases (see section 1.2.2), which can finally be linked to provide the complete NEPs’ SSbD case. To enable SSbD implementation, the knowledge derived through the analysis of the NM/NEPs entire LC needs to be handled according to the FAIR principles to secure its access and use in the long run, allowing the reproducibility of the analysis. Data curation is by far the most major issue addressed, regarding its central role in nanoinformatics, workflow, data completeness, and quality (Karcher et al., 2018; Papadiamantis et al., 2020; Furxhi et al., 2020; Jeliazkova et al., 2021). Experimental quantitative and computational derived data will be captured, curated, and used for optimization purposes prior to analysis (Furxhi, 2022; Furxhi et al., 2021a). FAIRified primary data are stored in the project’s database^10^ (ASINA-DB) where data logging templates, specific to data generation, are designed and populated for each working package and partner. Efforts have been put together with all the relevant stakeholders to capture the data in a harmonized manner, for example, to consent on the metrics used in quantitative data or importance of features regarding categorical variables (Furxhi, 2022; Furxhi et al., 2021a; Furxhi et al., 2021b; Furxhi et al, 2021c). The methodological and transparent data capturing approach allows the integration of data across NM/NEPs’ LC.

The ASINA-SMM faces the following challenges:

-Data harmonization and integration of information through NM/NEPS entire LC: Data alignment and integration scheme of the information derived from NM/NEPs’ LC is required for the results to be representative. To enable such a challenge, the entire consortium is informed and aligned with the FAIR principles, where data are enriched with metadata descriptions to allow their integration in a transparent manner. Multiple inner communication among the consortium are performed in a monthly basis, to avoid potential data gaps.

-Characterization during entire LC for Material-SSbD (M-SSbD): SSbD approach to materials/products starts with defining the design hypothesis based on intended use, production technologies, and known quality, in alignment with customer demands and industrial perspective on sustainability, safety, and functional performance. In this category characterization, toxicological, functionality, and synthesis-related cost data are generated as sub-categories. The selected NMs are kept constant during the subsequent information flow. The physicochemical (p-chem) properties are key starting points not only for risk assessments of NMs but also for formulating design hypothesis for products, in response to performance requirements. Briefly, extensive characterization is performed within ASINA to generate high-quality data across the entire LC of the NMs, since it is well documented that p-chem properties alter during their lifespan depending on the environmental or biological compartment (Mattsson and Simkó, 2017; Oomen et al., 2018).

Hazard for the NMs/NEPs is identified through *in vitro* testing (from simple monocultures to complex 3D co-culture models) representative of the main human exposure routes and the toxicological assessments will be based on an Adverse Outcome Pathway Tiered approach (Halappanavar et al., 2021). The ibid study established a “tissue injury” key event and showed how available literature with its limitations can be used to assess its biological plausibility; in addition, the methodology is available through the NanoCommons project, with which ASINA has initiated a Transnational Access scheme. Various toxicological assessments have been performed, targeting multiple target organs, such as skin corrosion test (OECD TG 431), skin irritation test (TG 439), Neutral Red Uptake - inflammatory mediators, cytotoxicity with MTT test/Alamar Blue for viability, colony forming efficiency (CFE), cytostatic index, mRNA expression/Protein expression, *in vitro* micronucleus test (OECD 487), and transepithelial resistance.

In addition, proper exposure data (from field exposure monitoring campaigns and the estimation of the internal dose using tools such as Multiple-Path Particle Dosimetry model) have been selected to define appropriate *in vitro* dosimetry ranges and dose metrics (Schmid and Cassee, 2017).

Functionality is defined by antimicrobial, photocatalytic, anti-aging, and antioxidant tests on the NEPs such as antibacterial finish textile materials (bacterial reduction, %, AATCC100-2012), determining the antimicrobial activity of antimicrobial agents (NMs) under dynamic contact conditions (ASTM E2149-13), photocatalytic efficiency of textiles and NMs (%), encapsulation efficiency (%) of cosmetics, antioxidant tests (DPPH method, %) of cosmetics, quantification of active ingredients (HPLC-UV) in cosmetics, etc.

The expected improvement of the environmental and economic sustainability is based on LCA and LCC analyses. LCA is developed according to the standard (ISO:14040-14044) by using Ecoinvent 3.8 database with impact assessment CML 2001. LCC is carried out in parallel by considering the same functional unit employed for the environmental studies. It has to be considered that ASINA-ES (see section 1.2.2) is an LCA/LCC software and database independent and can be operated to support the SSbD by providing suitable input data or through data available in databases related to the design case studies.

-Exposure estimation uncertainty under Process-SSbD (P-SSbD): P-SSbD approach applied to nanomanufacturing aims at minimizing occupational exposure and improving sustainability through a considerable reduction of consumes and environmental impacts, in compliance with safety requirements of occupational scenarios.

In this category release, fate and internal estimation exposure-related data are generated to address safety during usage and end of life. Various methodologies will be used such as textile abrasion methods (ISO 12947-2:2016), rubber- or plastics-coated fabrics (ISO 5470-1:2016), toxicity characteristic leaching procedure (EPA standard Method 1311;167), dissolution rates in diverse environmental and biological compartments, and bioaccumulation tests.

In addition, environmental/occupational assessment data are generated to address safety processes via measurement campaigns and exposure modeling parameterization for occupational safety decision-making (Furxhi et al., 2021c; Koivisto et al., 2021). Various direct-reading instruments (DRIs), such as SMPS, OPS, CPC, and personal monitors, as well as personal samplers for off-site analysis, will be used to monitor process airborne emissions and occupational exposure. Uncertainty is tackled by the variety of instrumentation, but the challenge then shifts to the intercomparison of the various instruments/techniques and the correct interpretation to derive exposure scenarios. The exposure assessments will be based on workplace exposure—Assessment of exposure by inhalation of nano-objects and their aggregates and agglomerates (EN 17058:2018) and workplace exposure—Measurement of exposure by inhalation to chemical agents—Strategy for testing compliance with occupational exposure limit values (EN 689:2018). The data associated with process airborne emissions and worker exposure are core information for the design and development of innovative P-SSbD solutions based on Digital Twins (Digital twin framework for manufacturing ISO 23247). LCC and LCA data are also generated for sustainability purposes of the processes.

-Ensuring regulatory framework compliance. ASINA identifies p-chem properties, and the variations due to intended applications and processes through an extensive characterization strategy (OCED ENV/JM/MONO (2019) that determines hazard potential of NPs/NEPs across the LC fulfilling regulatory requirements (REACH, OECD TGs, biocides, and cosmetics) such as

1) chemical substance composition and structure information (i.e., based on absorption and diffraction methodologies: x-ray and electron diffraction), label-free imaging of active molecules in nanostructured cosmetic materials, etc.,
2) particles’ physical properties [i.e., morphology, polydispersity, size distributions, and zeta potential through EM (electron microscopy) including TEM (transmission EM) and SEM (scanning EM), energy dispersive x-ray spectroscopy], and
3) surface properties (such as surface area calculated from gas adsorption, based on the Brunauer-Emmett-Teller method and Coherent anti-stokes Raman Spectroscopy (CARS) and resonance-enhanced surface second-harmonic generation (SHG).

ASINA outputs interface with reference regulatory requirements and integrate safety-assessment methods and approaches in line with existing standardization deliverables and authoritative documents (ISO/TC 229, CEN/TC 352, OCED, NIOSH and SCCS guidelines).

The SSbD actions consist of the parameterization and tuning of production procedures, materials selection, etc., based on the real data derived from the pilot actions of the two value chains products. The goal of these actions is to maximize safety while optimizing efficacy by comparing materials and process design solutions. However, it should be pointed out that sometimes it is not feasible to maximize both dimensions at the same time (Soeteman-Hernandez et al., 2019). Optimization requires iterations to reach a balance of efficacy and safety (Hjorth et al., 2017). For this reason, a metamodel tool that considers the factors affecting safety and functionality and quantitatively simulates the impact in each iteration of application is built.

#### 1.2.2 ASINA-Expert System

**FIGURE 2:**
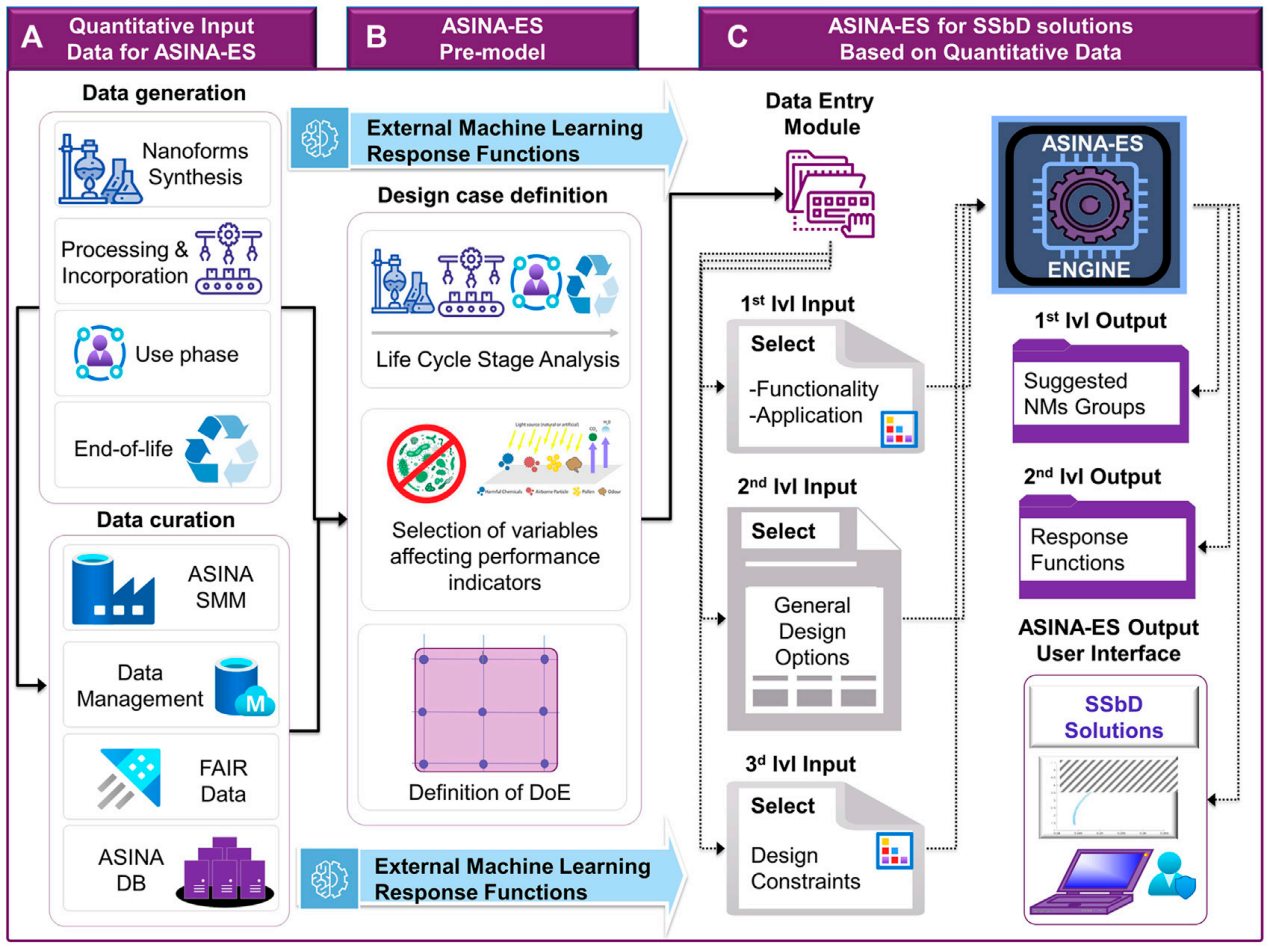
ASINA-ES metamodel elaborating response functions as representation of experimental or computed performances for the selection of SSbD solutions.

ASINA-ES is a multifactorial analysis configuration that uses suitable algorithms for minimization or maximization of response functions. It transfers outputs (set of ranked M-SSbD and P-SSbD solutions) for their validation through pilot action. The solutions are referred to a defined set of quantifiable *performance indicators* (functional, economic, environmental, and safety performances). Each design case corresponds to a point in the *design space* (Figure 2), whose coordinates represent the dominant variables affecting the performance indicators, and which may determine a better design option by varying their set points. The design case point coordinates are provided by the NEP user/designer with the quantitative values of the independent variables associated to the selected SSbD case performances.

The set of performance indicators define the dimensions of the *performance space*. The ranges of applicable variables values are specified, and based on modeling and pre-computation, a suitable *Design of Experiment (DoE)* domain is defined in the design space (Figure 2). Each point of the DoE mesh represents a defined design case characterized by specific coordinate values assigned to relevant variables within the corresponding allowed range. The ASINA-ES platform will be equipped with computational kernel (ES engine), data repository, and user interface and will return back to the user quantitative selected data for synthesis and processing set values as well as selected suitable options for use and end-of-life (disposal/recycling/reuse) phases according to possible circularity schemes.

**FIGURE 3:**
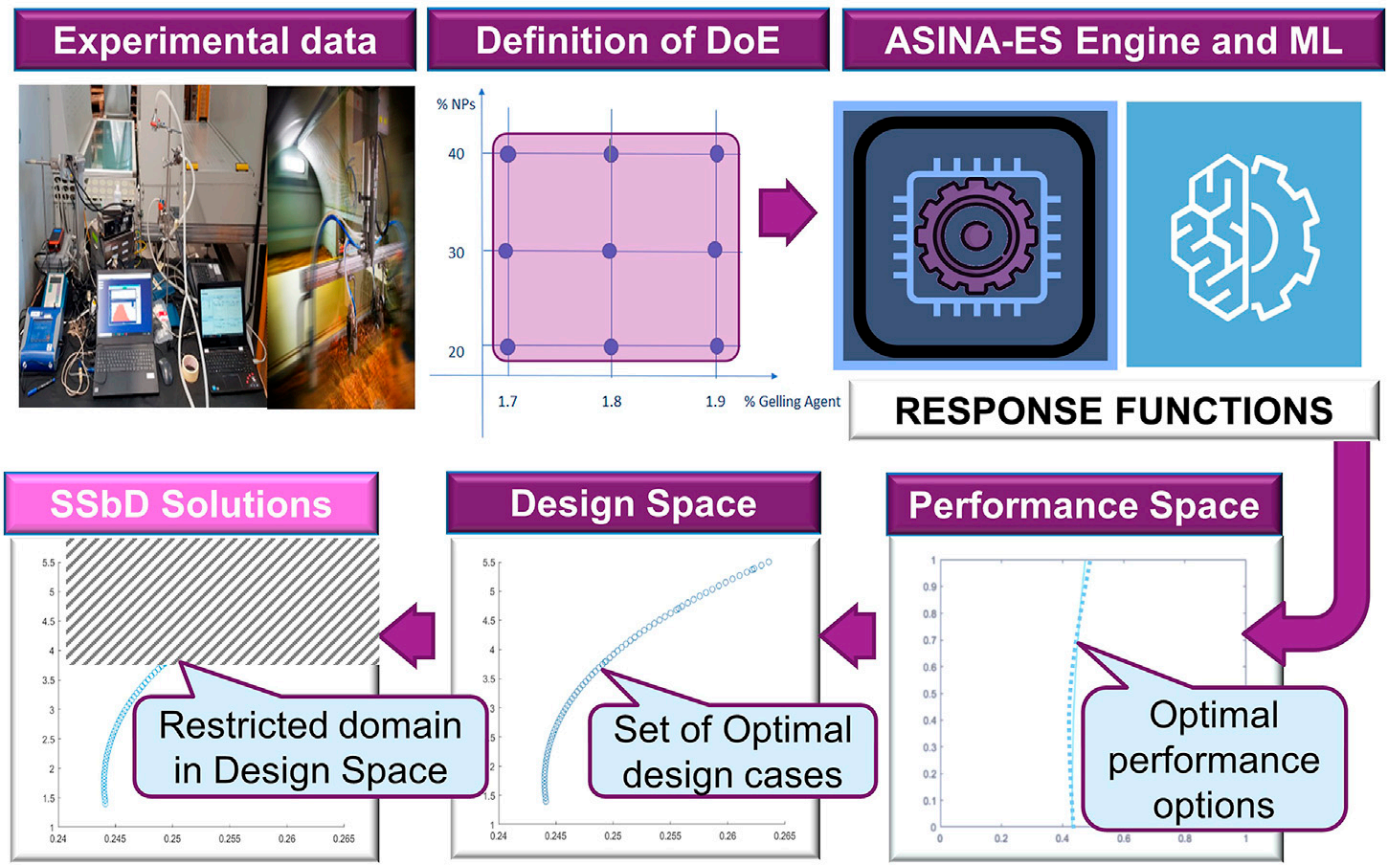
The ASINA-ES modular architecture is based on **(A)** quantitative data, elaboration, and FAIRification of experimental data and their **(B)** further processing through the ASINA-ES platform and external machine learning regressors, which derive the response functions. **(C)** Based on the computational kernel (engine), the set of SSbD solutions are computed and reported through the ASINA-Graphical Users Interface (GUI). The suggested set of alternative solutions is a valuable support to the NEP designers’ decisions process based on quantified expected performance in terms of product functionality, environmental and economic sustainability, as well as safety through the NEPs’ LC Stages.

In the first step, ASINA-ES processes the FAIRified data (Figure 3) using experimentally derived data from the pilot actions, which lead to the definition of the DoE, as well as machine learning regressors data, to find out the functional dependence between the independent values of the dominant variables and the associated performance indicators, thus providing the response functions (Figure 3). The response functions are complex representations of the NEP’s functional, environmental, economic, and safety performance indicators and are evaluated within the specific domain of the design space, referred to the specific LC phase (synthesis; incorporation, use and end of life). Only allowed solutions are selected as optimal SSbD alternatives.

In a second step, after loading the response functions objects to define the performance response space, the computational kernel performs a multi-objective optimization process in order to identify a sub-set of points in the design case space. This allows restricting the set of all feasible design case options complying with the independent variable’s domain constraints. This is obtained through the implementation of a customized Multi Criteria Decision Model (MCDM) that uses suitable algorithms for simultaneous minimization and maximization of all the response functions (Figure 3).

Finally, the SSbD options are presented to the user in terms of attainable best performances cases in the performance space. The tool is equipped with a (1) GUI and input/output modules to facilitate external data import and user interaction in order to define new design cases, recall developed designed cases, and update, compare, and interpret optimization results; (2) DB that will include the optimization results from ASINA case studies; and (3) ASINA-ES engine composed of computational modules for elaborating input data and implementing multi-criteria optimization algorithms to obtain the optimal set of sustainable performance. A set of SSbD solutions is returned to the user providing quantitative and measurable outputs, in terms of points in the performance space. The user will be able to select among the suggested sets of SSbD cases, which is/are the one(s) better fitting the design requirements. The ASINA-ES modular architecture also enables a flexible and comprehensive design process, by allowing interconnecting synthesis and incorporation processing options.

## 2 Discussion

The key features of the proposed data-driven approach is the collection and FAIRification of data, the machine learning application that links the data to performance indicators, and the integration of MCDM employed in the industrial process and product engineering. The holistic approach taking into consideration the safety, environmental, functional, and cost dimensions through the MCDM will guarantee that the SSbD solutions will achieve sustainability levels. This is a new paradigm for a quantitative-based SSbD approach for the development of competitive NEPs complying with regulations and safety requirements by also leveraging effective industrial uptake of nanotechnologies solutions. The approach is a technology enabler ensuring a faster acceptance of the industrial uptake of nanotechnology, translating the outputs of the research into tangible impacts on society, on human health, and the environment. In particular, the ASINA-ES

- is flexible as it may be applied to address multi-functionalities by defining suitable performance indicators of the NEP development and helping to identify the best route to devise the design strategy.
- leverages the NEP designer potential as it provides evidence of the simultaneous effects of a specific design choice by reducing the experimental and characterization burden, strongly decreasing the time to market and the NEP development costs.
- provides freedom for a multiscale modeling and assessment by iteratively refining the subdomains in the design space that allow selecting the best design options.

The ASINA-SMM is a data-driven approach containing all the important elements to consider to integrate safety early and throughout the development of NEPs. It can as well be applied to other types of applications beyond the specific industrial cases addressed within ASINA, after modification of data requirements. Furthermore, ASINA-ES is designed to host an indefinite number of design case studies, involving different synthesis and processing technologies, different products targeted to any application domain and use, as well as different options for managing the end of life of NEPs. The most important variables that span around pillars can be determined, leading to few parameters to be fed in ASINA-ES. In current times, where data curation and quality are becoming of paramount importance, we envisage that adequate magnitudes of high-quality data will become available and accessible, scaling up the applicability of the tool in diverse fields.

Industrial product design processes imply devising solutions for attaining competitive products possessing better functionalities and reduced manufacturing and operational costs along with assuring product compliance with standards and regulations. ASINA-ES is easily adoptable for industries as a valuable tool supporting NEPs design according to SSbD criteria, which, on top of the cost and functionality analysis, includes the environmental and nano-safety dimensions. Indeed, ASINA-ES is structured according to a multi-level modular framework that allows developing independent SSbD case studies, each referred to a specific NEP LC phase. Therefore, in the NEP whole design process, performance indicators related to nanoforms synthesis, processing and incorporation, use phase, and end-of life options are considered to globally qualify and quantify the SSbD profile of the addressed NEP.

## Data Availability

The original contributions presented in the study are included in the article/Supplementary Material. Further inquiries can be directed to the corresponding authors.

## Footnotes

1
https://www.sabyna.eu/ *(Accessed September 2021)*

2
https://www.sabydoma.eu/ *(Accessed September 2021)*

3
https://www.sbd4nano.eu/ *(Accessed September 2021)*

4
https://www.harmless-project.eu/ *(Accessed September 2021)*

5
https://www.h2020sunshine.eu/ *(Accessed September 2021)*

6
https://echa.europa.eu/regulations/nanomaterials/nanomaterials-expert-group *(Accessed September 2021)*

7
https://www.nanosafetycluster.eu/international-cooperation/the-malta-initiative/ *(Accessed September 2021)*

8
https://www.nanosafetycluster.eu/ *(Accessed September 2021)*

9
*Primary SbD approach developed within the EU projects NANoREG and NanoReg2, and the ProSafe initiative for the field of NEPs*

10
https://cloud.asina-project.eu/apps/files/?dir=/&fileid=4446
